# Enzymatic Disruption of Biofilms During Cheese Manufacturing: A Mini Review

**DOI:** 10.3389/fmicb.2021.791061

**Published:** 2021-12-16

**Authors:** Murali Kumar, Joseph Tierney, Martin Wilkinson

**Affiliations:** ^1^Department of Biological Sciences, University of Limerick, Limerick, Ireland; ^2^Glanbia Ingredients, Kilkenny, Ireland

**Keywords:** biofilms, EPS, cheese manufacturing, enzymes, CIP

## Abstract

Bacteria are capable of colonizing industrial processing surfaces creating biofilms on them which may adversely affect the quality and safety of products. Traditional cleaning-in-place (CIP) treatments using caustic and nitric acid solutions have been known to exhibit variable efficiency in eliminating biofilm bacteria. Here, we introduce enzymes as an alternative to traditional CIP treatments and discuss their mechanism of action against bacterial biofilms in cheese manufacturing. In addition, we discuss research gaps namely thermal stability, substrate specificity and residual activity of enzymes that may play a vital role in the selection of enzymes with optimal effectiveness against multi species biofilms. The outcome of this mini review will aid in the development of a novel and sustainable enzyme-based CIP treatment during cheese manufacturing in the future.

## Introduction

In the dairy industry including cheese manufacturing, bacteria colonize processing surfaces through biofilms which may adversely impact the quality and safety of milk and cheese products by contamination (Bremer et al., 2009; Sadiq et al., 2016).

To combat biofilms, the dairy industry uses CIP methods involving circulation of cleaning solutions containing 0.5–2% caustic soda and 0.5–1% nitric acid at high velocity with turbulent flow at elevated temperatures (Seale et al., 2010; Thomas and Sathian, 2014). Previous studies have indicated that the effectiveness of CIP treatments can vary in eliminating surface adhered biofilms, raising the need for an alternative treatment with a higher reliability (Faille et al., 2001; Marchand et al., 2012). Bremer et al., 2006, studied the effectiveness of different CIP treatments and reported a large variation in the ability of sodium hydroxide to consistently remove dairy biofilms. This finding was in accordance with previous studies that have reported such variations (Flint et al., 1999; Dufour et al., 2004). Several factors including the age, composition of the biofilm, cleaning time and temperature have been previously identified to influence the effectiveness of CIP treatments (Changani et al., 1997; Lelievre et al., 2001).

Recent advancements have highlighted the role of enzymes in replacing the caustic-based cleaning agents with potential benefits including reduced energy and water consumption, environmental impact and improved safety (Boyce et al., 2010; Delhalle et al., 2020). Previous studies have primarily focused on enzyme-based strategies to combat biofilms in the medical and food industry except cheese manufacturing (Nahar et al., 2018; Saggu et al., 2019; Jiang et al., 2020). In this mini review, we provide an overview on the role of enzymes as an alternative CIP treatment and their mechanism of action on biofilms during cheese manufacturing which has not been discussed previously. In addition, we highlight some research gaps in the use of enzymes as a potential CIP agent during cheese manufacturing. This information will benefit future investigations on the underlying mechanism of action of enzymes on biofilms in the dairy industry.

## Biofilm Formation in a Cheese Manufacturing Plant-The Underlying Problem

**FIGURE 1:**
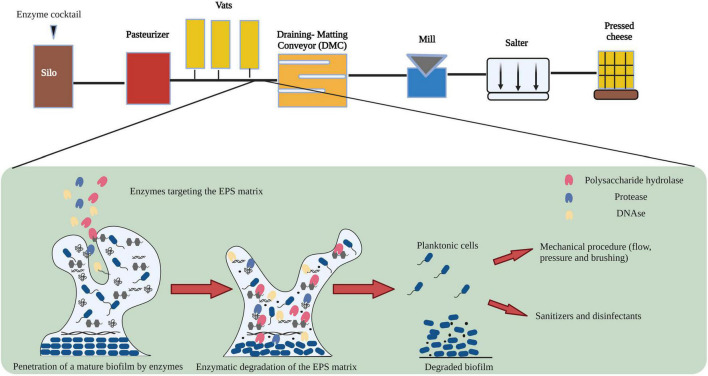
Flow diagram showing the general mechanism of action of enzymes on biofilm formed during cheese manufacturing. Image adapted from Nahar et al. (2018) and Johnson et al. (2021) with permission and was re-created with the BioRender software.

Johnson et al., 2021, demonstrated variation among microbial sub populations within sections of cheese manufacturing environments. The process flow diagram of cheese manufacturing is shown in Figure 1. Raw milk stored in silos at low temperatures is prone to microbial contamination resulting from the formation of biofilms by heat sensitive *Pseudomonas* and *Listeria* species (Shaheen et al., 2010; Marchand et al., 2012). In addition, Zhang et al., 2020, demonstrated that psychotropic bacteria are capable of growing and forming biofilms during storage of refrigerated raw milk. Raw milk can be contaminated with heat stable enzymes produced by a broad spectrum of psychotropic bacteria which may survive heat processing steps and can affect the quality of dairy products including cheese (Vithanage et al., 2016). Following storage in silos, raw milk is pasteurized to reduce the population of planktonic cells including spoilage microorganisms (Visser and Jeurnink, 1997). However, vegetative cells and spores of psychrotrophic, mesophilic and thermophilic bacteria are capable of surviving such treatments, germinating, and forming biofilms within the plate heat exchanger (PHE) (Meer et al., 1991; Flint et al., 2002; Hinton et al., 2002) or on processing equipment (Kumar et al., 2021). Equipment surfaces in the draining and matting conveyor (DMC) are made of stainless steel which is a known substrate for biofilm formation by psychrotrophic, mesophilic and thermophilic bacteria (Suarez et al., 1992; Zhao et al., 2013; Sadiq et al., 2017; Kumar et al., 2021). In addition to stainless steel pipelines, the accumulation of food borne pathogens in the form of biofilms on rubber gaskets and seals made of Buna-N and Teflon have been discussed previously (Austin and Bergeron, 1995; Kumar and Anand, 1998). In the design of an enzyme-based CIP treatment, it is vital to consider the spatial, temporal variability of microbial sub populations at different sections of the cheese manufacturing plant and the varying chemical composition of biofilms which consequently demands an optimized enzyme combination for their removal.

## Mechanism of Action of Enzymes on Biofilms

**FIGURE 2:**
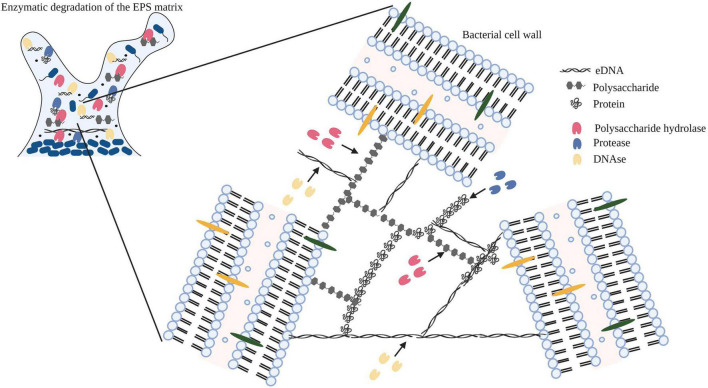
Diagrammatic representation of the mechanism of action of enzymes (polysaccharide hydrolase, protease, and DNase) targeting exopolysaccharides, proteins, and eDNA within the EPS matrix. Image has been adopted from Nahar et al. (2018) with permission and was recreated with BioRender software. Black arrows indicate the binding of the enzyme to the substrate.

In bacterial biofilms, the extracellular matrix plays a vital role in the establishment and maintenance of the biofilm structure and is composed primarily of extracellular polymeric substances (EPS) (Flemming and Wingender, 2010; Di Martino, 2018). The EPS matrix is composed of proteins, polysaccharides, and nucleic acids (eDNA and RNA) along with lipids and ions (Kristensen et al., 2008; Whitfield et al., 2015; Coughlan et al., 2016). Previous studies have identified a strain related variation in the composition of polysaccharides in the EPS matrix (Banik et al., 2007; Torres et al., 2012; Roca et al., 2015; Zhang et al., 2015). In addition to strain variation, several other factors can potentially influence the yield and composition of the EPS matrix (Li et al., 2021). Lequette et al., 2010, analyzed the cleaning efficiency of polysaccharidase and proteolytic enzymes against biofilms of bacterial species isolated from the food industry and concluded that the composition of the EPS matrix affected the choice and cleaning efficiency of the enzyme treatment. In Figure 2, we demonstrate the mechanism of action of an enzyme cocktail containing polysaccharide hydrolase, protease, and DNase on a mature biofilm matrix.

Earlier studies have discussed the effect of alkaline and acidic cleaning agents on the biofilm matrix which differs in comparison with enzymes (Liikanen et al., 2002; Parkar et al., 2004; Dogsa et al., 2005). The substrate specificity of enzymes may contribute toward their enhanced efficiency for the removal of biofilms in comparison with alkali and acid cleaning agents and needs further investigation. In addition, the design of an enzyme-based CIP treatment should proceed in tandem with developing good hygiene practices of equipment, surfaces, and devices.

## Gaps and Future Recommendations

Studying the potential advantages and shortcomings in the use of enzymes for CIP treatment is important for designing a robust cleaning regime with a higher cleaning efficiency compared to conventional CIP treatments.

Thermal stability of enzymes plays a vital role in determining the enzymatic activity which may impact the efficiency of the CIP treatment (Boyce et al., 2010). These authors demonstrated that three commercial proteases were inactivated by any of the heat sanitation steps commonly employed in the dairy industry. Future research should further investigate the thermal stability of several commercially available enzymes and consider the same when designing an effective CIP treatment. Lequette et al., 2010, who screened the biofilm removal ability of seven proteases and polysaccharidase concluded that the efficiency of enzymes on biofilm removal depended on the bacterial species with proteases being more efficient in removing *Bacillus* biofilms in contrary to polysaccharidase being more effective against *Pseudomonas fluorescens* biofilms of industrial origin. To our knowledge, the use of enzymes for CIP does not imply any associated effects on bacterial viability within biofilms. Further research is warranted to study the substrate specificity of enzymes to increase the range of action of an enzyme treatment against multi species who form complex biofilms of varying compositions. Besides the thermal stability and substrate specificity, the residual activity of an enzyme is a critical parameter that may impact the selection criteria for enzymes to be used in CIP treatments. Boyce et al., 2010, concluded that an acid circulation step (0.5–1% nitric acid at 60°C) is capable of inactivating the residual enzyme activity of ten commercial proteases and lipases, remaining on the processing equipment surface after cleaning. In the cheese industry, residual enzyme activity may impact the quality of cheese through bitterness generation, excessive proteolysis and lipolysis and hence processing steps are needed to ensure complete inactivation of any added enzyme used for CIP. Furthermore, the impact of enzymes on the dairy processing wastewater treatment needs to be evaluated and future steps should focus on the recovery of enzymes from dairy processing wastewater sludge (DPS). Future research on the development of an enzyme-based CIP treatment for cheese manufacturing in the European Union must comply with the expectations on the use of enzymes in the food industry as per regulations (EC) No 1332/2008 and (EC) No 648/2004.

In addition to enhanced cleaning efficiency, enzyme-based cleaning is associated with lower rinsing volumes which results in reduced water consumption and water costs (Boyce et al., 2010). Moreover, the wastewater generated from an enzyme-based cleaning may require minimal processing due to lack of need for a neutralization step involving the lowering of the pH of the effluent stream which may also lower the operating cost (D’Souza and Mawson, 2005).

## Conclusion

The implementation of an enzyme-based cleaning as an alternative to traditional CIP treatment is favorable due to the improved cleaning performance provided there is no deterioration to the product quality. In this review, we highlight the mechanism of action of enzymes used as a CIP treatment, however, further studies are needed to study the substrate specificity and thermal stability of commercially available enzymes that can be used as cleaning agents in cheese manufacturing.

## Author Contributions

MK conducted the literature review and wrote the manuscript. JT and MW edited and revised the manuscript. All authors contributed to the article and approved the submitted version.

## Conflict of Interest

JT is employed by Glanbia Ingredients, Ireland. The remaining authors declare that the research was conducted in the absence of any commercial or financial relationships that could be construed as a potential conflict of interest.

## Publisher’s Note

All claims expressed in this article are solely those of the authors and do not necessarily represent those of their affiliated organizations, or those of the publisher, the editors and the reviewers. Any product that may be evaluated in this article, or claim that may be made by its manufacturer, is not guaranteed or endorsed by the publisher.
